# Interaction Between Angiotensin-Converting Enzyme Gene Insertion/Deletion Polymorphism and Angiotensin-Converting Enzyme Inhibition on Survival in Hemodialyzed Patients

**DOI:** 10.1097/MD.0000000000000315

**Published:** 2014-12-02

**Authors:** István Kiss, Csaba Ambrus, Imre Kulcsár, János Szegedi, Lóránt Kerkovits, András Tislér, Zoltán Kiss

**Affiliations:** From the B.Braun Avitum Hungary (BRAVHU) CPLC Dialysis Network (IK, CA, IKulcsár, JS, LK, BBAVHU-DIALGENE Workgroup); Department of Nephrology-Hypertension, St Imre University Teaching Hospital, Budapest (IK, CA, LK); Division Section of Geriatrics, 2nd Department of Internal Medicine, Semmelweis University Faculty of Medicine, Budapest (IK); School for Ph.D. Candidates of Aesculap Academy, Budapest (IKulcsár, ZK); and 1st Department of Internal Medicine, Semmelweis University Faculty of Medicine, Budapest, Hungary (AT).

## Abstract

The association between ACE (angiotensin-converting enzyme) gene insertion/deletion (I/D) polymorphism and mortality has been inconsistently observed in earlier studies in patients on maintenance hemodialysis. We hypothesized that the effect of ACE gene I/D polymorphism on mortality may be influenced by concurrent ACE inhibitor therapy in this population.

In this prospective, multicenter cohort, observational study, data was collected from 716 prevalent chronic hemodialysis patients, blood samples were genotyped for I/D single nucleotide polymorphism. Patient mortality was assessed in tree genotype groups insertion/insertion, insertion/deletion and deletion/deletion (I/I, I/D, and D/D) using multivariate Cox proportional hazard models.

The most frequent genotype was I/D (42.6%), followed by D/D (37.7%) and I/I (19.7%) genotypes. The mean age was 54.9 ± 15.5 years, 53.2% of all patients were male and in the total group the prevalence of diabetes was 19.3%. ACE inhibitor therapy was prescribed for 47.9% of all patients. The median duration of dialysis before blood sampling was 23.8 months (IQR 11.2–47.1). Patients were followed for 10 years, the median follow-up time was 29.8 months (IQR 12.6–63.4). Patient characteristics were well balanced among the genotype groups. D/D genotype, was associated with inferior survival (I/I vs D/D: log-rank test: *P* = 0.04) in patients not receiving ACE inhibitor therapy, and the presence of this therapy diminished this difference. There was no difference in survival among unselected patients with different genotypes. In multivariate Cox regression models, D/D genotype (compared to I/I) was a significant predictor of mortality only in patients without ACE inhibitor therapy (HR 0.67, 95% CI 0.46–0.97, *P* = 0.03).

Our data suggests that hemodialyzed patients with the deletion/deletion (D/D) genotype might have inferior outcome, and ACE inhibitor therapy may be associated with improved survival in this subgroup.

## INTRODUCTION

While during the past decade, the trend in the growing number of prevalent patients on renal replacement therapy (RRT) started to level off and after initiation of RRT the survival time is increasing in Europe, managing chronic kidney disease (CKD) related health conditions remain a difficult challenge.^1^ The mortality of dialyzed CKD stage 5 (CKD-5D) patients is high in comparison to the general population^2^ and the leading cause of death continues to be of cardiovascular origin.^3^ One of the cardiovascular risk factors in this population may be the high concentration and activity of angiotensin-converting enzyme (ACE). The concentration of this enzyme is significantly determined by the ACE gene insertion or deletion (I/D) polymorphism, which is responsible for the wide variance of inter-individual enzyme activity. Individuals with the deletion/deletion (DD) genotype have approximately 2 times higher ACE concentration in comparison to individuals with insertion/insertion (I/I) genotype.^4^ As a consequence, this polymorphism was considered to contribute to the high prevalence of cardiovascular morbidity and death^5^ and also to decreased survival time in CKD patients once dialysis is initiated.^6^ Indeed, several authors found an association between risk of mortality and the ACE gene I/D genotype. In patients with renovascular atherosclerotic disease, the longest survival time was observed when subject had I/I genotype followed by the insertion/deletion (I/D) genotype and there was significantly shorter survival in patients with D/D genotype. Furthermore, D/D genotype was a significant predictor of mortality independently of other risk factors in multivariate models.^7^ Also in patients with type 2 diabetes and diabetic nephropathy, increased mortality was associated with the D/D genotype. The authors suggested that genotyping CKD patients for this polymorphism would be useful in clinical practice so that health care providers could select patients with higher mortality risk early and start an intensive medical care.^8^ In a Dutch multicenter prospective study, 453 dialyzed patients were followed up for 4 years. The mortality risk was the highest among patients with the D/D genotype.^9^ On the other hand, some studies in CKD patients could not prove the association between the ACE gene I/D polymorphism and cardiovascular diseases as a leading cause of death.^10,11^ Results of a previous meta-analysis from the turn of the millennium were also conflicting.^12^ A later meta-analysis, however, found a positive association between the D allele and coronary artery disease.^13^ It seems, therefore, that the question regarding the effect of ACE gene on survival continues to be unanswered. Furthermore, the conflicting previous results raise the question whether there were other parameters that may influence or interact with the effect of the ACE gene I/D polymorphism on mortality in clinical trials. One of the influencing factors could be the pharmacology blockade of the renin-angiotensin-aldosterone system (RAAS). Indeed, we have previously shown that ACE inhibitor therapy can influence the genetic effect of this polymorphism^14^ on erythropoietin resistance in hemodialysis CKD stage 5 (CKD-5HD) patients. In addition, we also claimed further research to clarify the association between ACE activity, inhibition and mortality.^15,16^

The aim of this study, therefore, was to assess the association between long-term survival and ACE gene I/D polymorphism in CKD-5HD patients. Furthermore, we aimed to determine whether concurrent ACE inhibitor therapy influenced the effect of ACE gene I/D polymorphism on mortality. Our hypothesis was that the D/D genotype associated with inferior outcome compared to the I/I genotype and concurrent ACE inhibitor therapy can influence the higher mortality in patient with the D/D genotype.

## PATIENTS AND METHODS

All eligible patients, who were dialyzed in 11 centers of B.Braun Avitum Hungary CPLC Dialysis Network, were enrolled into the study in 1997. Inclusion criteria were a minimum of 91 days on dialysis at the time of cross-sectional data capture and written informed consent to the study. Seven hundred forty-six dialyzed CKD patients were eligible for enrolment into the observational study, and their baseline data were collected. The time of start of dialysis was collected retrospectively and then patients were followed prospectively for 10 years between 1997 and 2007 and the database closing was March 31, 2007. During this prospective follow-up period, we collected data only for mortality. Thirty patients had incomplete dataset and, therefore; they were excluded from the analysis. Data collected retrospectively and prospectively from 716 patients were analyzed. After obtaining written informed consent, we captured baseline demographic, clinical data and a blood sample for genetic testing. Survival time was calculated as a total survival time, from the date of initiation of RRT (dialysis vintage at study start plus follow-up period) for each patient.

Ethylenediaminetetraacetic acid (EDTA) samples were collected before dialysis and DNA was isolated from peripheral blood leukocytes by standard non-enzymatic method. From peripheral blood samples, the genotyping was performed by PCR technique.^17^

Parameters following a normal distribution are reported as mean and standard deviation (SD). Categorical variables are presented as absolute frequency and percentages. For further statistical analysis, we grouped patients by the presence of ACE inhibitor therapy, genotype and age. Age was calculated at the time of cross-sectional data collection. Comparison of groups was performed by Student's *t*-test or ANOVA (Tukey-test used for post-hoc analysis) or Kruskal–Wallis, and Mann–Whitney tests for continuous variables and *z*-test for categorical variables. Survival analysis was done using Kaplan–Meier product-limit model, and we reported median and 95% CI values of survival time. Survival in different genotype groups was compared using log-rank test. Potential predictors of survival were analyzed in multivariate Cox proportional hazard models, and we reported hazard ratios (HRs) and 95% confidence intervals (95% CIs). *P*-values with a 2-sided alpha of 0.05 were considered statistically significant. Statistical analysis was performed by STATISTICA software package (version 10. Tulsa, OK) and MedCalc software (version 13.1, Ostend, Belgium).

All patients gave written consent to the genetic testing and data collection. The study was approved by the local and central Ethical Committee, and it was financially supported by the Hungarian Scientific Research Fund (OTKA, TO23927) and B.Braun Avitum Hungary CPLC Dialysis Network. This research adhered to the principles of the Declaration of Helsinki.

## RESULTS

Baseline characteristics of patients by genotype are presented in Table 1. The majority of the patients had I/D genotype (n = 305) followed by the D/D (n = 270) and lastly by the I/I (n = 141) genotypes. The different parameters were well balanced among the groups. There was only 1 significant difference, proportion of patients with hypertension as a cause of renal disease was significantly higher in the I/I group (*P* = 0.02). The dialysis therapy of 194 patients (27.1%) was started within 1 year before the data capture. The majority of individuals, 400 patients (55.9%) within 1 to 4 years, 104 patients (14.5 %) within 5 to 9 years and 18 patients (2.5 %) started their dialysis therapy more than 10 years before the data capture.

**TABLE 1:**
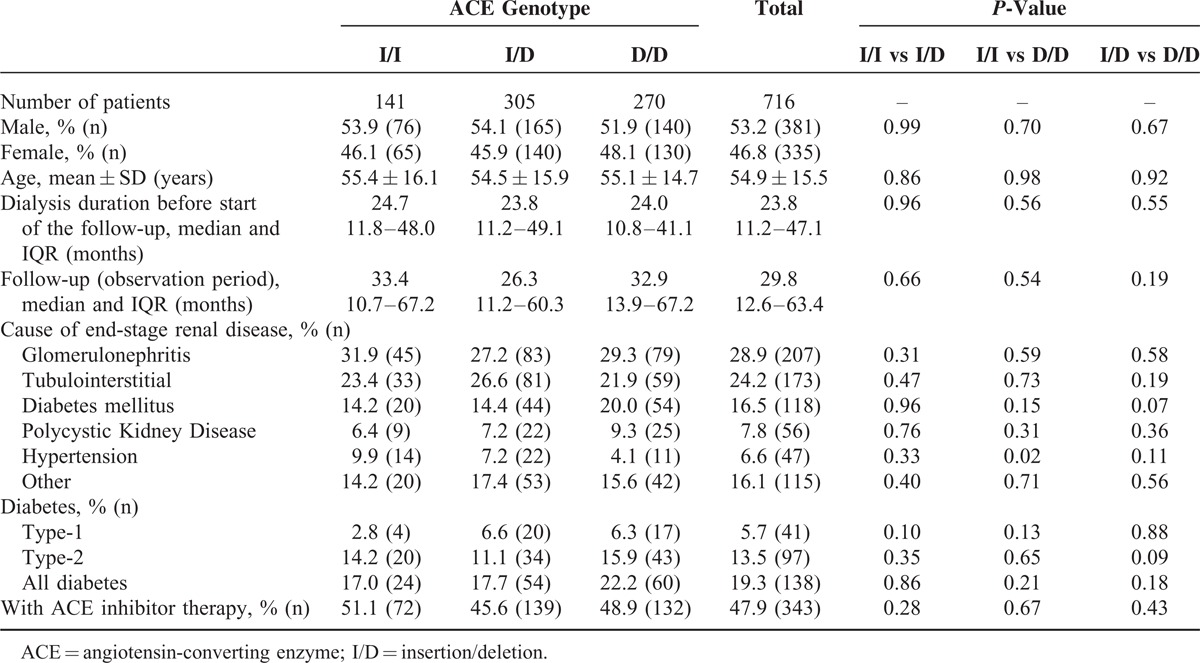
Baseline Characteristics of Dialyzed Patients by Genotype

**FIGURE 1:**
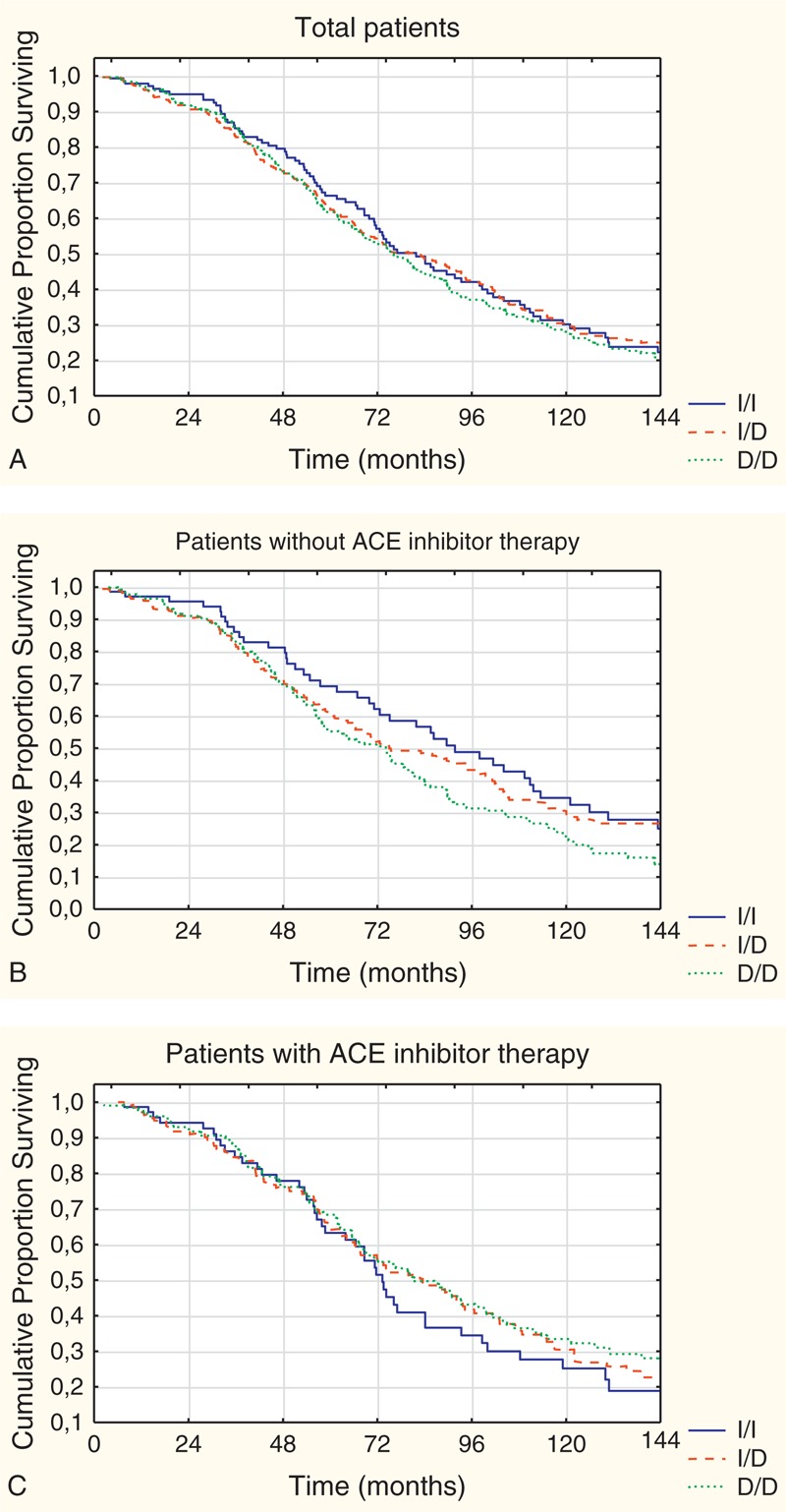
(a) Kaplan–Meier survival estimates for patients with different genotypes (log-rank test: *P* = 0.69). (b) Kaplan–Meier survival estimates for genotype groups in patients without ACE inhibitor therapy (I/I vs D/D: log-rank test: *P* = 0.04) (c) Kaplan–Meier survival estimates for genotype groups in patients with ACE inhibitor therapy (log-rank test: *P* = 0.81).

Overall, 62.7 percent of patients died during the 10-year follow-up period. Among all patients, including retrospective and prospective periods with a maximum of 144 months follow-up, survival was not different among the different genotype groups (Figure 1a). The rate of all cause mortality was similar in the genotype groups. The median survival time was 81.9 (95% CI 71.2–98.6) months in patients with I/I genotype, 82.9 (95% CI 68.0–94.3) months with I/D genotype and 76.1 (95% CI 67.4–86.6) months with D/D genotype.

**FIGURE 2:**
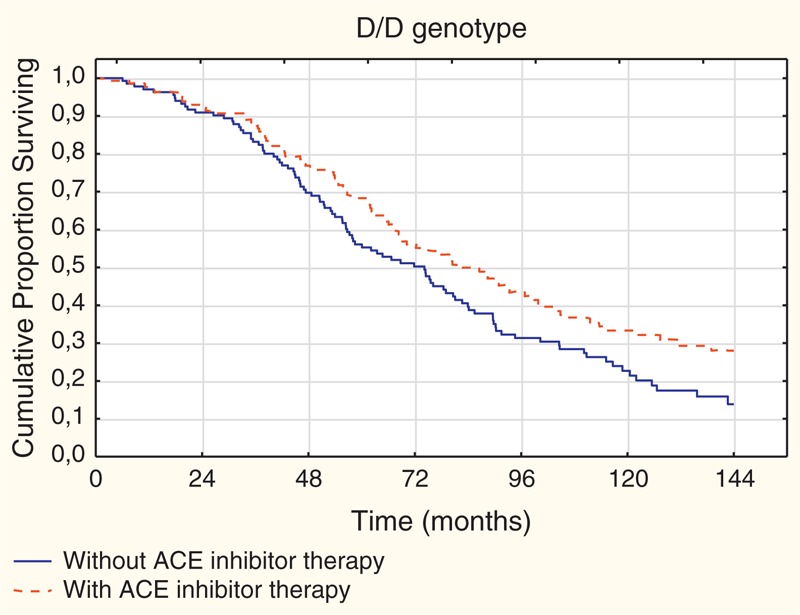
Kaplan–Meier survival estimates for patients with versus without ACE inhibitor therapy in patients having D/D genotype (n = 270; without ACE inhibitor therapy n = 138; with ACE inhibitor therapy n = 132) (with vs without the therapy: log-rank test: *P* = 0.03).

After stratification of patients by the presence or absence of ACE inhibitor therapy, we observed different results. While in the subgroup without ACE inhibitor therapy mortality rate was higher in patients with the D/D genotype (I/I vs D/D: log-rank test: *P* = 0.04), in the other subgroup with ACE inhibitor therapy the difference was not significant (Figure 1b and c). In the subgroup without ACE inhibitor therapy, the survival time was significantly (*P* = 0.04) shorter in patients with D/D genotype (median: 74.2/95% CI 57.3–82.7/months) than in patients with I/I genotype (median: 91.7/95% CI 71.2–111.7/months). Further analysis revealed that in patients with the D/D genotype the presence of ACE inhibitor therapy was associated with significantly improved survival as compared to patients, without ACE inhibitor therapy (Figure 2) (*P* = 0.03). In this D/D genotype subgroup, patients with ACE inhibitor therapy had longer survival time (median: 81.4/95% CI 68.4–99.8/months) than their counterparts without ACE inhibitor therapy (median: 74.2/95% CI 57.3–82.7/months) significantly (*P* = 0.03).

In multivariate Cox proportional hazard model, the genotype was not a significant predictor of mortality neither in the whole patient group nor patients with ACE inhibitor therapy (Table 2). In contrast, in patients without ACE inhibitor therapy the ACE D/D genotype became a significant predictor of death (vs the I/I genotype, *P* = 0.03). Age and diabetes were significant predictors of survival in all 3 subgroups (*P* < 0.001 for all models). Concerning dialysis duration before start of the follow-up (12 months increments) was not significant (*P* = 0.91) predictor of survival in Cox proportional hazard model (data not shown).

**TABLE 2:**
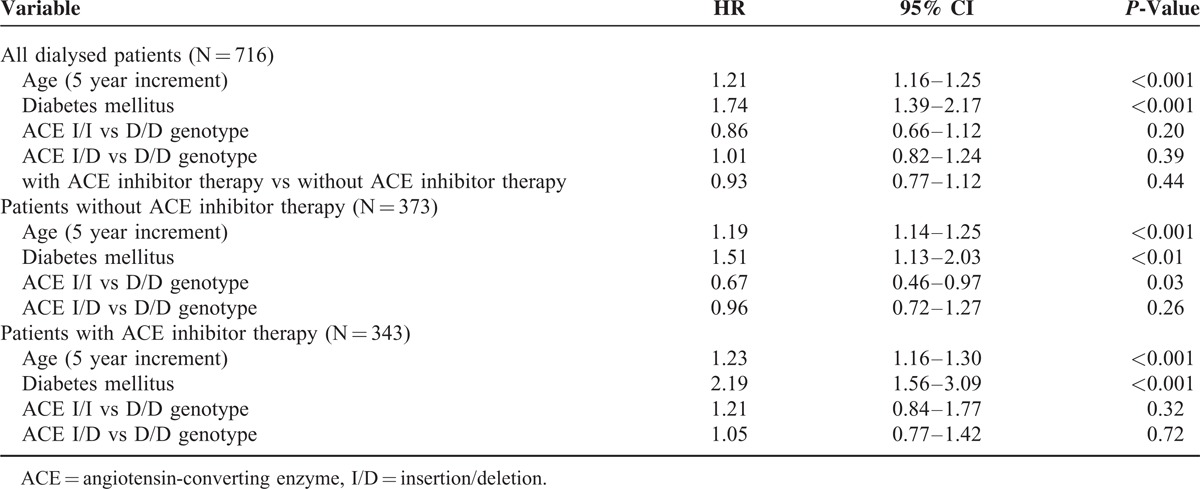
Multivariate Cox Proportional Hazard Models for Survival

## DISCUSSION

In this study, we demonstrated that the D/D genotype is a risk factor of mortality in hemodialysis patients without ACE inhibitor therapy. In contrast, we could not find an association between the ACE gene I/D polymorphism and survival among unselected CKD-5HD patients during long-term follow-up. Our results suggest that the effect of this polymorphism is in fact, a risk factor for mortality, and that this genetic effect may be influenced by concomitant ACE inhibitor therapy. This interaction could explain for the conflicting results of previous studies.

To our knowledge, we are the first to demonstrate that among hemodialyzed CKD patients without ACE inhibitor therapy, the survival time is significantly shorter in patients with the D/D genotype compared to those with the I/I genotype. This relevant difference in survival disappeared in patients with ACE inhibitor therapy. As a result, among patients with the D/D genotype the unfavorable genetic and favorable ACE inhibition effects on survival time are undoubtedly obvious. When the genetic effect was undisturbed the mortality rate was high while during pharmacological blockade of the RAAS cascade, this polymorphism could not manifest its effect on survival. Based on these results we propose a strong causal relationship between the genetic effect of ACE gene I/D polymorphism on mortality as well as the clinical manifestation of the gene on survival and pharmacological inhibition of RAAS cascade. The latter one seems to have a profound influence on the clinical manifestation of ACE gene polymorphism.

These results of our study are in concordance with other works in which the D-allele was found to be a risk factor in hemodialysis CKD patients.^7–9,13^ On the other hand, there is no contradiction to previous trials, which could not find, such an association, as—to our knowledge—none of them considered the effects of RAAS inhibition. The lack of stratification of patients by the presence or absence of ACE inhibition in previous trials may have obscured the effect of the D/D genotype on survival, and explain for the contradictory results. With the present work demonstrating the pivotal role of the genetic factor on mortality in hemodialysis CKD patients, genetic testing of patients in the future may help us to better evaluate mortality risk and contribute to risk stratification in this population. Nevertheless, further studies are required to evaluate the beneficial role of ACE or RAAS inhibition on survival time of dialyzed patients with different genotypes.

There are several limitations of our work. The observational nature of the study does not allow to elucidate clear causal effect. In addition, the follow-up time of almost all enrolled patients consists of retrospective and prospective parts because the vast majority of patients were enrolled when they already had been on dialysis. This could result in negative selection of patients who died between initiation of dialysis and data capture. However, sensitivity analyses including patients with variable duration of dialysis before data collection revealed similar survival results (data not shown). In addition, the duration of dialysis was longer in patients with I/I genotype not receiving ACE inhibitor compared to the D/D genotype. These observations further support our hypothesis. Regarding ACE inhibitor therapy, we had no data on the duration and persistence of therapy, and whether patients commenced such treatment after baseline data collection. However, stopping or starting ACE inhibitor therapy after the data capture would have biased our results toward finding no associations. Furthermore, we did not analyze effect of angiotensin-1 receptor blockers because during the observation period, these newer RAAS inhibitor therapies were not yet available in Hungary. In addition, we do not have data about the patient's co-morbidities, which may influence the mortality in the different groups. Finally we have not data about serum ACE activity in the different genotype subgroups. Despite all those limitations, we believe that our results are valid, and the revealed associations prove our hypothesis.

## CONCLUSION

Our results confirm that in CKD-5HD patients, without ACE inhibitor therapy the D/D genotype is associated with inferior outcome compared to the I/I genotype. In addition, ACE inhibitor therapy is associated with improved survival in CKD-5HD patients with the D/D genotype. However, the ACE gene I/D polymorphism has no effect on survival in an unselected cohort of CKD-5HD patients. As a result, hemodialysis CKD patients with the D/D genotype may benefit from ACE inhibitor treatment. Further studies exploring the effect of ACE gene polymorphism on clinical outcomes should consider the presence or absence of concomitant RAAS inhibitor therapy.
